# Epidural Venous Plexus Engorgement: What Lies Beneath?

**DOI:** 10.1155/2015/965106

**Published:** 2015-02-04

**Authors:** Fuldem Yildirim Donmez

**Affiliations:** Department of Radiology, Baskent University Medical Faculty, Fevzi Cakmak Caddesi, Bahcelievler, 06490 Ankara, Turkey

## Abstract

Epidural venous plexus engorgement may occur due to several conditions that prevent the normal venous circulation. Inferior vena cava agenesis is a very rare cause of epidural venous enlargement. We present a case with a very thin inferior vena cava and left iliac vein agenesis who presented with back pain due to epidural vein engorgement and lacked other venous problems such as deep vein thrombosis.

## 1. Introduction

Back pain and nerve root compression symptoms are very frequent complaints of patients with herniated discs which are demonstrated on lumbar MRI. However, herniated discs may not be the only pathology and MRI may also reveal epidural venous engorgements which cause nerve root compression. In that case, underlying etiological factors must be thought in the differential diagnosis such as absence of inferior vena cava, intracranial hypotension, and Marfan syndrome [1]. Engorgement of the epidural veins may be observed as patent dilated veins, thrombosed dilated veins, or submembranous, epidural-contained hematoma on MRI [2].

Herein we present a young patient with back pain who had epidural venous engorgement on lumbar MRI. Subsequent abdominal CT revealed a very thin inferior vena cava and absence of left common iliac vein.

## 2. Case Report

**Figure 1:**
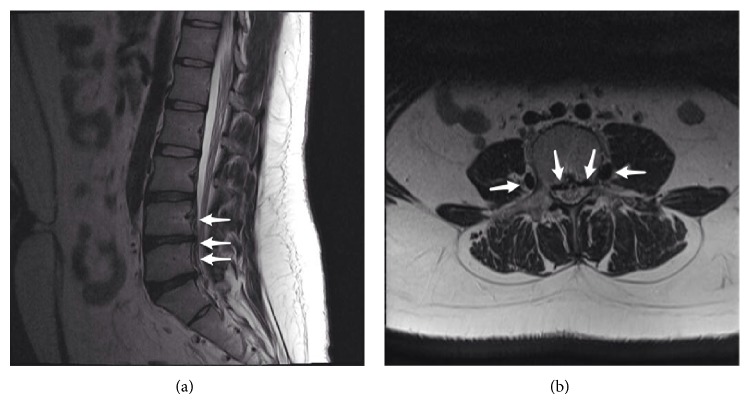
Sagittal T2-weighted image shows vascular enlargement in the epidural area at the level of lower lumbar vertebrae (a). Axial T2-weighted image shows the epidural veins obliterating the epidural space and engorgement of the ascending lumbar vein (b).

**Figure 2:**
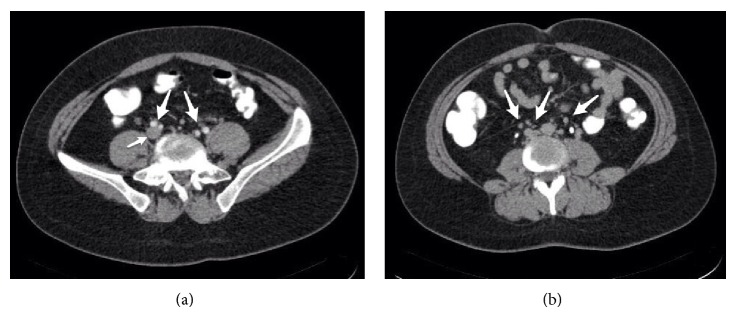
Axial CT image shows the patent left and right internal and external iliac arteries (arrows) and the patent right common iliac vein (small arrow) (a). Axial CT image from a lower level shows the collateral vascular structures (arrows) (b).

A 24-year-old woman with back and left leg pain was admitted to the department of neurosurgery. She mentioned that the back pain was refractory to medical treatment. She had no prior medical problems except for polycystic ovaries and irregular menstruation periods. Physical examination revealed the Lasegue positivity at 45 degrees. A lumbar MRI with the prediagnosis of disc herniation was requested. On lumbar MRI, no herniation was evident; however there were venous engorgement of the epidural veins and obliteration of the anterior epidural space on axial and sagittal T2-weighted images (Figures 1(a) and 1(b)). The patient was sent for an abdominal CT with the question of any pathology that may prevent venous circulation. Contrast-enhanced abdominal CT showed that the inferior vena cava was very thin and left common iliac vein was absent (Figure 2(a)). The azygos and hemiazygos were enlarged. There were collateral vascular structures in the paravertebral and epidural areas and renal hili (Figure 2(b)). She had no prior complaint related to leg pain or any findings of vein thrombosis. Family history for thrombosis was negative. In genetic investigation, a Factor V Leiden mutation was found. There was no prothrombin mutation. She was given prophylactic antithrombotic treatment.

## 3. Discussion

Epidural venous engorgement may be seen in blocked venous system due to various pathologies such as portal hypertension, Budd-Chiari syndrome, intracranial hypotension, superior or inferior vena cava thrombosis, and abdominal malignancy or in a physiologic state such as pregnancy. It may be asymptomatic, or in case of nerve root compression, there may be radicular symptoms [3, 4]. The symptoms may not be due to direct nerve root compression from distended veins. Venous congestion may cause radiculopathy through ischemia [5]. Priorly, arterial impairment was thought to be the main responsible factor of neuroischemia; however it is now known that venous circulation may also play a role in spinal ischemia that may cause radiculopathy [6].

Thrombosis of inferior vena cava due to several diseases, some of which are mentioned above, is more common. Absent inferior vena cava is a very rare cause of this disorder. Normally developed inferior vena cava is formed by anastomosis of several embryological segments. Absence of suprarenal segment results in continuation of venous flow into the azygos system Congenital aplasia of the infrarenal segment of IVC itself is extremely rare [4, 7]. Even though it may be asymptomatic, it is also one of the causes of deep vein thrombosis especially in young patients, so incidentally caught cases should be cautioned for thrombosis.

Radiologically it is important to be aware of other structures rather than disc pathology which may cause radicular symptoms. In a case report by Dudeck et al., the dilated veins were thought to be retroperitoneal enlarged lymph nodes on MRI and ultrasound revealed multinodular left-sided retroperitoneal mass which later turned out to be thrombosed collateral veins [7]. Radiologic differential diagnosis may be challenging when the dilated veins are more localized. Herniated disc, hematoma, abscess, tumor, and synovial cyst must be thought in the radiologic differential diagnosis [1]. Epidural venous engorgement is more common in the lumbar region; however cervical root compression due to cervical epidural venous plexus enlargement which mimicked an epidural mass was also reported [8]. Treatment of the enlarged epidural veins which are symptomatic may be either antithrombotic medical drugs such as heparin or surgical treatment such as venous thrombectomy [1]. Endovascular treatment with vena cava stenting was applied to a patient who had intractable radicular and low back pain secondary to inferior vena cava stenosis associated with Budd-Chiari syndrome [9].

Generally published cases are presented with findings of thrombosis, and inferior vena cava agenesis without thrombosis is reported in an article by Kamerath and Morgan which became symptomatic during exercise [4]. Our patient also did not have any finding of thrombosis. She had only back pain and mild left leg pain. The first reason is thought as the disc herniation, stenosis of the neural foramen, or spinal canal clinically.

The cases in the literature took place in clinical journals rather than radiology journals; however, we think that radiologists should be aware of this phenomenon and in such cases immediate abdominal workup for any vascular pathology and also doppler sonography should be performed to rule out possible deep vein thrombosis. Diagnosis of inferior vena cava or iliac vein agenesis or thrombosis will also help to lead the antithrombotic treatment and also knowing any Factor 5 Leiden mutation is also important in young women who may be pregnant and have the possibility of thrombosis-related problems in the future. This problem is not confined to the lumbar region and ascending lumbar vein and epidural venous plexus enlargement should be sought in the cervical or thoracal regions.
